# Proton Pump Inhibitor Usage and the Risk of Myocardial Infarction in the General Population

**DOI:** 10.1371/journal.pone.0124653

**Published:** 2015-06-10

**Authors:** Nigam H. Shah, Paea LePendu, Anna Bauer-Mehren, Yohannes T. Ghebremariam, Srinivasan V. Iyer, Jake Marcus, Kevin T. Nead, John P. Cooke, Nicholas J. Leeper

**Affiliations:** 1 Stanford Center for Biomedical Informatics Research, Stanford University, Stanford, CA, United States of America; 2 Department of Cardiovascular Sciences, Houston Methodist Research Institute, Houston, TX, United States of America; 3 Practice Fusion, Inc., San Francisco, CA, United States of America; 4 Divisions of Cardiovascular Medicine and Vascular Surgery, Stanford University, Stanford, CA, United States of America; University of Louisville, UNITED STATES

## Abstract

**Background and Aims:**

Proton pump inhibitors (PPIs) have been associated with adverse clinical outcomes amongst clopidogrel users after an acute coronary syndrome. Recent pre-clinical results suggest that this risk might extend to subjects without any prior history of cardiovascular disease. We explore this potential risk in the general population via data-mining approaches.

**Methods:**

Using a novel approach for mining clinical data for pharmacovigilance, we queried over 16 million clinical documents on 2.9 million individuals to examine whether PPI usage was associated with cardiovascular risk in the general population.

**Results:**

In multiple data sources, we found gastroesophageal reflux disease (GERD) patients exposed to PPIs to have a 1.16 fold increased association (95% CI 1.09–1.24) with myocardial infarction (MI). Survival analysis in a prospective cohort found a two-fold (HR = 2.00; 95% CI 1.07–3.78; P = 0.031) increase in association with cardiovascular mortality. We found that this association exists regardless of clopidogrel use. We also found that H_2_ blockers, an alternate treatment for GERD, were not associated with increased cardiovascular risk; had they been in place, such pharmacovigilance algorithms could have flagged this risk as early as the year 2000.

**Conclusions:**

Consistent with our pre-clinical findings that PPIs may adversely impact vascular function, our data-mining study supports the association of PPI exposure with risk for MI in the general population. These data provide an example of how a combination of experimental studies and data-mining approaches can be applied to prioritize drug safety signals for further investigation.

## Introduction

The primary indication for proton pump inhibitors (PPIs) is gastroesophageal reflux disease (GERD). Each year, it is estimated that over 113 million PPI prescriptions are filled globally. This, together with over-the-counter use, accounts for over $13 billion sales worldwide [1] [2]. In the US alone, about 21 million people used one or more prescription PPIs in 2009, making it the third highest seller in the country [3][2]. The availability of PPIs over-the-counter is particularly more worrisome due to the absence of medical supervision [1].

For individuals with a history of acute coronary syndrome (ACS), PPIs appear to reduce the efficacy of clopidogrel, an antiplatelet agent used to reduce the risk for subsequent ischemic events [4]. There are several competing theories about whether (and how) PPIs enhance the risk of major adverse cardiovascular events (MACE) amongst individuals with a history of ACS.[5–10] A leading hypothesis is that PPIs compete for and inhibit the clopidogrel-activating hepatic isoenzyme, CYP2C19, thereby interfering with clopidogrel’s capacity to prevent clot formation in subjects at risk for coronary thrombosis and myocardial infarction (MI).[11]

However, some studies have associated PPI usage with adverse clinical outcomes in high-risk cardiovascular populations, independently of clopidogrel use.[7] For example, a reduction in therapeutic benefit has been reported in ACS patients treated with the antiplatelet agents aspirin and ticagrelor, neither of which requires activation by CYP2C19. [12, 13] While it is possible that PPIs may reduce the absorption of these drugs (a controversial hypothesis given that PPIs have been shown not to diminish the anti-platelet aggregation properties of aspirin [14, 15]), it is important to note that a similar reduction in gastric pH is achieved with H_2_ blockers (H_2_Bs), which have been shown not to increase cardiovascular risk [12, 13].

An alternative explanation is that the observed risk of PPIs is due to some unknown mechanistic pathway [12], and that this pathway may not be restricted to vasculopathic patients. In this regard, we recently reported that PPIs inhibit the enzymatic activity of dimethylarginine dimethylaminohydrolase (DDAH), [16] which is responsible for 80% of the clearance of asymmetric dimethylarginine (ADMA)—an endogenous molecule known to inhibit the enzymatic activity of nitric oxide synthase (NOS).[17] An impairment in endothelial NOS (eNOS) is well-known to increase vascular resistance, and promote inflammation and thrombosis.[18] ADMA is a potent disease marker and independent predictor of MACE in prior observational studies.[19–24] Our recent pre-clinical studies found that PPIs increase ADMA levels in human endothelial cells and in mice by about 20–30%.[16]

To date, we are aware of only one study which has examined the cardiovascular risk association of PPIs outside of high-risk cohorts [25]. This is a concern given our translational data, which suggests that the risk of these drugs may apply to subjects not taking antiplatelet agents, and those without any vascular disease. Therefore, we employed a novel and recently validated [26, 27] data-mining approach for pharmacovigilance on multiple electronic medical record datasets as well as examined a prospectively followed clinical cohort [28, 29], to explore the possibility that PPIs may be associated with cardiovascular risk in the general US population.

## Methods

The data mining studies were deemed by the Stanford IRB not to involve human patients. The Stanford GenePAD study was approved by the Stanford Human Subjects Research Institutional Review Board and was conducted under the guidelines of the Declaration of Helsinki, with written informed consent was obtained from all participants.

### Data sources

We used two data sources for our data mining analysis—a primary source from Stanford and a secondary source from Practice Fusion, Inc—and one prospective source for the survival analysis.

At Stanford University, all clinical notes (both inpatient and outpatient) have been transcribed and recorded electronically since 1994. These data are warehoused for research use in the Stanford Translational Research Integrated Database Environment (STRIDE).[30] STRIDE contains data from 1.8 million patients, 19 million encounters, 35 million coded International Classification of Disease (ICD-9) diagnoses, and a combination of pathology, radiology, and transcription reports totaling over 11 million unstructured clinical notes.

Practice Fusion, Inc. (PF) provides a free, web-based Electronic Health Record (HER) system for clinicians. The company’s users are primarily small practices providing outpatient care. Roughly, half of these practices specialize in primary care, with 29% of users from the West, 13% from the Southwest, 14% from Midwest, 27% from the Southeast, and 18% from the Northeast. The de-identified subset of PF data used in our analysis contained data on 1.1 million patients, 5.5 million coded diagnoses, 6.8 million prescriptions, and 5.5 million unstructured clinical notes dating back to 2007.

Additionally, we examined the association of PPI use at enrollment with subsequent cardiovascular mortality in the GenePAD (the Genetic Determinants of Peripheral Arterial Disease) [28, 29] study. The GenePAD cohort is comprised of individuals who underwent an elective, non-emergent coronary angiogram for angina, shortness of breath or an abnormal stress test at Stanford University or Mount Sinai Medical Centers. Cardiovascular mortality was defined as that from myocardial infarction, cardiac arrest, stroke, heart failure or aneurysm rupture. Cardiovascular outcomes were assessed through medical record review and confirmed by contacting the patient or next of kin directly. This form of dual follow-up was specifically implemented to limit detection bias from differential frequencies in physician contact between groups. Finally, all deaths were confirmed and cross-referenced to the SSDI to minimize detection bias. The study cohort commenced in 2004 and included 1,503 individuals.

### Data-mining pipeline for pharmacovigilance

We used a previously validated data-mining pipeline for pharmacovigilance using clinical data [26] [31] to screen whether the exposure to proton pump inhibitors is associated with an elevated risk of myocardial infarction in the general population. Note that such a data-mining procedure is not the same as performing an epidemiological study. The difference between performing an epidemiological study and a data-mining study is categorically described in [32]. Briefly, data-mining approaches focus on learning a valid function **f(x)**—which is modeled as an algorithm that operates on variables **(x)** to predict the responses **(y)**. The linking function f(x) in a data-mining study can be a regression, but cannot, and should not, be interpreted as a causal regression model which is typically the goal of an epidemiological study.

The validation of data-mining approaches is performed by measuring predictive accuracy and is widely adopted in computer science [33], and increasingly in economics [34]. Our data-mining approach, which aims to minimize false positives, has 97.5% specificity and 39% sensitivity in discerning a true association as determined using a gold standard set of 28 true positive and 165 negative associations spanning 78 drugs and 12 different outcomes [35]. This performance provides an accuracy of 89% and has a positive predictive value of 81% if we test an equal number of true and false associations. We summarize the approach briefly, and further details are provided in LePendu et al [26].

The pipeline extracted positive-present mentions of drug, disease, device, and procedure concepts from all clinical notes, accounting for negation and other contexts, into a patient–feature matrix that we analyzed. Drug terms were normalized to active ingredients using RxNorm, and classified according to the Anatomical Therapeutical Chemical classification system. For example, “Prilosec” and “omeprazole” were treated equally; while omeprazole, rabeprazole, and so on were grouped together as the class of PPIs. Disease terms were normalized and aggregated according to the hierarchical relationships from the Unified Medical Language System Metathesaurus and BioPortal. Finally, we aligned records temporally based on the time at which each note was recorded and only kept positive-present–first mentions. The matrix (for STRIDE) comprises nearly a trillion pieces of data—roughly, 1.8 million patients as rows, thousands of clinical concepts as columns, with time as the third dimension (see Fig 5 in LePendu et al [26]).

#### Patient population and outcome definition

GERD is the primary indication for PPIs, so we used the presence of this indication to define the baseline population in our pipeline. We excluded all patients under the age of 18 at their first GERD mention. We defined GERD by International Classification of Diseases, Ninth Revision (ICD-9) codes for esophageal reflux (530.81) and heartburn (787.1), and the UMLS code for gastroesophageal reflux disease (C0017168). The main outcome of interest, MI, was defined by acute myocardial infarction (ICD-9 code 410), and more than 18 different UMLS codes including myocardial infarction (C0027051) and silent myocardial infarction (C0340324). See S1 Table for full definitions.

#### Study groups and study periods

The study period included all data from 1994 through 2011 in STRIDE and 2007 through 2012 in PF. We defined two study groups within the GERD baseline population in this period. The primary study group was the subset defined by patients taking PPIs, including a sub-group of those patients who were not on clopidogrel. We considered six PPIs (omeprazole, lansoprazole, pantoprazole, esomeprazole, rabeprazole, and dexlansoprazole) individually and as a class. We excluded dexlansoprazole from individual analysis because of insufficient exposure (<100 patients). As an alternative treatment for GERD we examined H2 blockers (H2Bs—cimetidine, famotidine, nizatidine, and ranitidine) as a separate association test.

#### Association estimation

The summary of the data-mining pipeline shown in the S1 Fig outlines the decisions used in the data-mining pipeline to populate a contingency table for each of the associations tested. Each patient was counted according to the temporal ordering of concepts in the patient–feature matrix as described in LePendu et al [26]. For example, a mention of PPI use after a GERD indication would be counted as an exposure. A subsequent mention of MI counts as an associated outcome. Our data-mining method works based on “beforeness” of treatments and events and given the uncertainty the exact times of treatment and the messy EMR data used, we follow a two-step process for detecting drug safety signals (details in methods of LePendu et al) [26]. First we compute a raw association, followed by adjustment which involves matching on age, gender, race, length of observation, and, as proxies for health status, the number of unique drug and disease concepts mentioned in the full record. The first step is useful for flagging putative signals, and the second step in reducing false alarms. As in prior work, we attempted to match up to 5 controls. In cases where there are not enough controls to draw from, we tried either 1:3 or finally 1:1 matching (Table 1). The balance of variables before and after matching for the PPI study group is shown in Table 2. The balance of variables for the H2Bs study group is shown in Table 3. Note that the purpose of this matching is to reuse our validated two-step data-mining approach from LePendu et al [26] and not emulate an epidemiological study from the EMR data. In each of the two steps, we compute the odds-ratio as well as confidence interval (CI) using logistic regression and use a significance cutoff of p-value < 0.01.

**Table 1:** Study group populations for the STRIDE dataset, including 5:1 propensity matching.

		Before Matching			After Matching	
Study Group and Subgroups	N (GERD = 93,149)	Exposed	Control	Match Ratio	Exposed	Control
Age > = 18 ‡	70,477					
PPI *		32,363	38,114	1:1	32,363	32,099
(–) clopidogrel †		30,454	35,856	1:1	30,454	30,275
Age < = 45 * ‡		9,595	12,846	1:1	9,595	9,568
Age < = 55 * ‡		16,662	21,016	1:1	16,662	16,546
H_2_ blocker *		12,796	57,681	1:3	12,796	38,388
omeprazole *		8,921	28,498	1:3	8,921	26,763
esomeprazole *		2,907	27,049	1:5	2,907	14,535
pantoprazole *		4,450	28,001	1:5	4,450	22,250
rabeprazole *		2,473	26,972	1:5	2,473	12,365
lansoprazole *		4,005	27,596	1:5	4,005	20,025

There are 93,149 patients with gastroesophageal reflux disease (GERD), of which 70,477 are at least 18 years old. In all study groups other than the one in which clopidogrel patients are excluded, the populations are matched (and balanced) based on clopidogrel use and other covariates such as age, gender, race, length of observation, number of unique drugs mentioned in the record, and validated proxies of health status (based on the number of unique disease concepts attributed to each patient). The matching process attempts to achieve a ratio of 1:5 (exposure to control), but will settle for 1:3 or 1:1 if there are not enough controls from which to draw.

* balanced for clopidogrel use

† patients on clopidogrel are excluded

‡ patients are restricted by age

**Table 2:** Balance of variables for patients on PPIs in the STRIDE dataset.

Variable		Before Matching		After Matching	
	Exposed (N = 32,363)	Unmatched controls (N = 38,114)	p-value	Matched controls (N = 32,099)	p-value
**Demographics**					
Age at indication (GERD), mean (sd)	54.88 (16.65)	53.47 (17.15)	<2.22e-16	54.52 (17.13)	0.005
Gender (male), n (%)	44.31	45.60	0.0006	44.61	0.44
Race, n (%)					
Asian	7.93	8.92	< 0.0001	8.33	0.13
Black	2.77	2.84	0.62	2.96	0.15
Other	11.82	11.58	0.33	11.92	0.71
Unknown	27.07	26.52	0.09	25.84	0.0001
White	50.41	50.14	0.49	50.95	0.16
**Co-prescription**					
Clopidogrel, n(%)	5.90	5.93	0.89	6.16	0.16
**Other**					
**No. of unique diseases, n (%)**					
1–28	7.72	12.78	<0.0001	7.30	0.34
29–41	9.45	9.67	0.34	8.87	0.002
42–56	10.16	10.07	0.72	9.98	0.42
57–74	10.44	10.12	0.16	10.27	0.46
75–95	10.26	9.76	0.03	10.28	0.91
96–121	9.93	9.66	0.23	10.35	0.08
122–157	10.47	9.62	0.0001	10.79	0.18
158–207	10.37	9.37	9.9e-06	10.64	0.26
208–294	10.53	9.62	6.3e-05	10.79	0.26
>294	10.67	9.33	3.8e-09	10.73	0.81
**No. of unique drugs, n (%)**					
1–9	5.78	14.23	<0.0001	5.80	0.92
10–15	9.18	10.03	0.0001	9.48	0.16
16–22	11.05	10.48	0.01	10.89	0.50
23–29	9.76	8.92	0.0001	9.51	0.29
30–38	10.93	9.37	8.0e-12	10.66	0.27
39–49	10.74	9.84	0.0001	10.94	0.33
50–64	10.80	9.43	1.7e-09	10.93	0.60
65–86	10.81	8.95	2.2e-16	10.48	0.15
87–123	10.74	9.12	9.5e-13	10.61	0.57
>123	10.21	9.63	0.009	10.70	0.05
**Length of observation (days), n (%)**					
1–9	8.97	10.92	<0.0001	8.37	0.007
10–102	9.90	10.05	0.52	9.59	0.17
103–364	9.72	10.25	0.02	9.89	0.48
365–816	9.95	10.02	0.77	10.21	0.25
817–1426	9.87	10.11	0.29	10.12	0.21
1427–2156	9.64	10.31	0.003	10.10	0.05
2157–3018	9.77	10.20	0.06	10.27	0.03
3019–3956	10.40	9.66	0.001	10.48	0.76
3957–5084	10.88	9.25	8.4e-13	10.44	0.06
>5084	10.90	9.23	2.6e-13	10.53	0.12

PPI exposure and control groups for patients with gastroesophageal reflux disease (GERD) are balanced across a number of basic covariates. Importantly, the use of clopidogrel is equally balanced across exposed and control groups. Other variables (no. of unique disease mentions, no. unique drug mentions, length of observation) are binned according to their distributions. Before matching, PI patients tend to be sicker, have more diseases and more drugs mentioned in their record. After matching, these variables are balanced.

**Table 3:** Balance of variables in patients on H_2_ blockers in the STRIDE dataset.

Variable		Before Matching		After Matching	
	Exposed (N = 12,796)	Unmatched controls (N = 57,681)	p-value	Matched controls (N = 38,388)	p-value
**Demographics**					
Age at indication (GERD), mean (sd)	52.90 (16.85)	54.38 (16.95)	<2.22e-16	53.19 (16.74)	0.13
Gender (male), n (%)	42.76	45.50	1.38e-08	43.26	0.41
Race, n (%)					
Asian	9.27	8.28	0.0003	9.02	0.41
Black	3.41	2.68	2.55e-05	3.19	0.33
Other	11.80	11.67	0.67	11.60	0.61
Unknown	23.43	27.51	< 2.22e-16	24.24	0.12
White	52.09	49.86	4.70e-06	51.95	0.82
**Co-prescriptions**					
Clopidogrel, n(%)	6.99	5.67	7.1e-08	6.75	0.45
**Other**					
**No. of unique diseases, n (%)**					
1–28	6.05	11.44	< 0.0001	6.12	0.79
29–41	6.71	10.21	< 2.22e-16	6.83	0.68
42–56	7.92	10.60	< 2.22e-16	8.11	0.55
57–74	8.79	10.59	1.55e-10	9.22	0.21
75–95	9.14	10.17	0.0003	9.58	0.22
96–121	9.40	9.87	0.10	10.07	0.07
122–157	10.90	9.81	0.0003	11.88	0.01
158–207	11.46	9.47	9.59e-11	12.25	0.05
208–294	13.15	9.34	< 2.22e-16	13.19	0.92
>294	16.48	8.50	< 2.22e-16	12.75	< 2.22e-16
**No. of unique drugs, n (%)**					
1–9	4.01	11.83	< 0.0001	4.02	0.98
10–15	6.84	10.26	< 2.22e-16	6.78	0.85
16–22	8.33	11.25	< 2.22e-16	8.46	0.67
23–29	8.01	9.59	4.58e-09	8.24	0.48
30–38	9.91	10.12	0.47	10.68	0.04
39–49	10.37	10.20	0.58	11.18	0.03
50–64	10.90	9.87	0.0006	12.10	0.001
65–86	11.61	9.40	9.31e-13	12.63	0.008
87–123	13.29	9.10	< 2.22e-16	13.32	0.94
>123	16.73	8.38	< 2.22e-16	12.59	< 2.22e-16
**Length of observation (days), n (%)**					
1–9	6.56	10.81	< 0.0001	6.56	1
10–102	7.36	10.56	< 2.22e-16	7.40	0.90
103–364	8.45	10.35	7.87e-12	8.95	0.15
365–816	9.71	10.04	0.25	9.95	0.51
817–1426	9.97	10.01	0.91	10.24	0.47
1427–2156	10.44	9.90	0.07	10.61	0.65
2157–3018	10.68	9.84	0.005	10.70	0.97
3019–3956	11.80	9.60	1.17e-12	11.70	0.39
3957–5084	11.99	9.56	7.55e-15	11.69	0.46
>5084	13.04	9.33	< 2.22e-16	12.20	0.04

PPI exposure and control groups for patients with gastroesophageal reflux disease (GERD) are balanced across a number of basic covariates. Importantly, the use of clopidogrel is equally balanced across exposed and control groups. Continuous variables (no. of unique disease mentions, no. unique drug mentions, length of observation) are binned according to their distributions. Before matching, H_2_ blocker patients tend to be sicker, have more diseases and more drugs mentioned in their record. After matching, these variables are mostly balanced.

### Survival analysis in a prospective cohort

For all survival analyses in the GenePAD cohort, the follow-up time was defined as the period between the enrollment interview and the last confirmed follow-up or date of death. Cox proportional hazards models were used to calculate adjusted and unadjusted hazard ratios (HR) and 95% CI for the association of PPI use with cardiovascular mortality. Adjusted models included age, gender, race, total cholesterol, high-density lipoprotein cholesterol, systolic blood pressure, use of anti-hypertension medications, and lifetime pack-years.

## Results

Patients receiving clopidogrel (or other anti-platelet agents) post ACS have been extensively studied previously [5, 7–10, 12, 13, 15, 36]. In our study the primary population of interest is patients with GERD. We find that the class-level association of PPIs with MI in patients treated for GERD exists across two independent datasets and is independent of clopidogrel use and high-risk age groups. By comparison, we find no association with MI in GERD patients treated with H_2_Bs in the same dataset. The results from the data-mining effort are concordant with our analysis in a prospectively followed cohort from the GenePAD [28, 29] study, showing increased cardiovascular mortality associated with PPI use and no such increase associated with H_2_B use.

### Characteristics of the primary clinical dataset

All patients with GERD above the age of 18, representing the general population likely to take a PPI, comprise the baseline population for our studies. The two study groups include patients exposed to PPIs, and, for comparison, patients exposed to H_2_Bs. Controls were selected from the baseline population using propensity score matching [37] (see Methods).

Our results were replicated across two independent datasets—one from Stanford (~1.8 million patients) and a subset of data from Practice Fusion, Inc. (PF) (~1.1 million patients). Table 1 summarizes the characteristics of the baseline and study populations for the primary dataset from Stanford, called STRIDE. Similar distributions were seen in the PF dataset. Overall, out of all patients in STRIDE, 93,149 have had GERD (of which 70,477 are at least 18 years old); 22,411 have had a MI; 59,109 have taken at least one H_2_B; and 16,127 have taken clopidogrel. The characteristics of each of the study groups are balanced for exposed and unexposed patients, noting in particular that clopidogrel use is balanced (Tables 2 and 3).

In the baseline population for STRIDE (N = 70,477), 45.9% used at least one PPI (12.7% omeprazole, 5.7% lansoprazole, 6.3% pantoprazole, 4.1% esomeprazole, 3.5% rabeprazole, and 0.1% dexlansoprazole) and 18.2% used an H_2_B (Table 1). The mean follow-up time is 2.1 years in the PPI study group, and 2.5 years in the H_2_B group. Of all PPI patients, less than 6% used clopidogrel—highlighting the relatively small size of the well-studied ACS populations compared to the general population of PPI users.

### A safety signal for an association with MI

**Fig 1:**
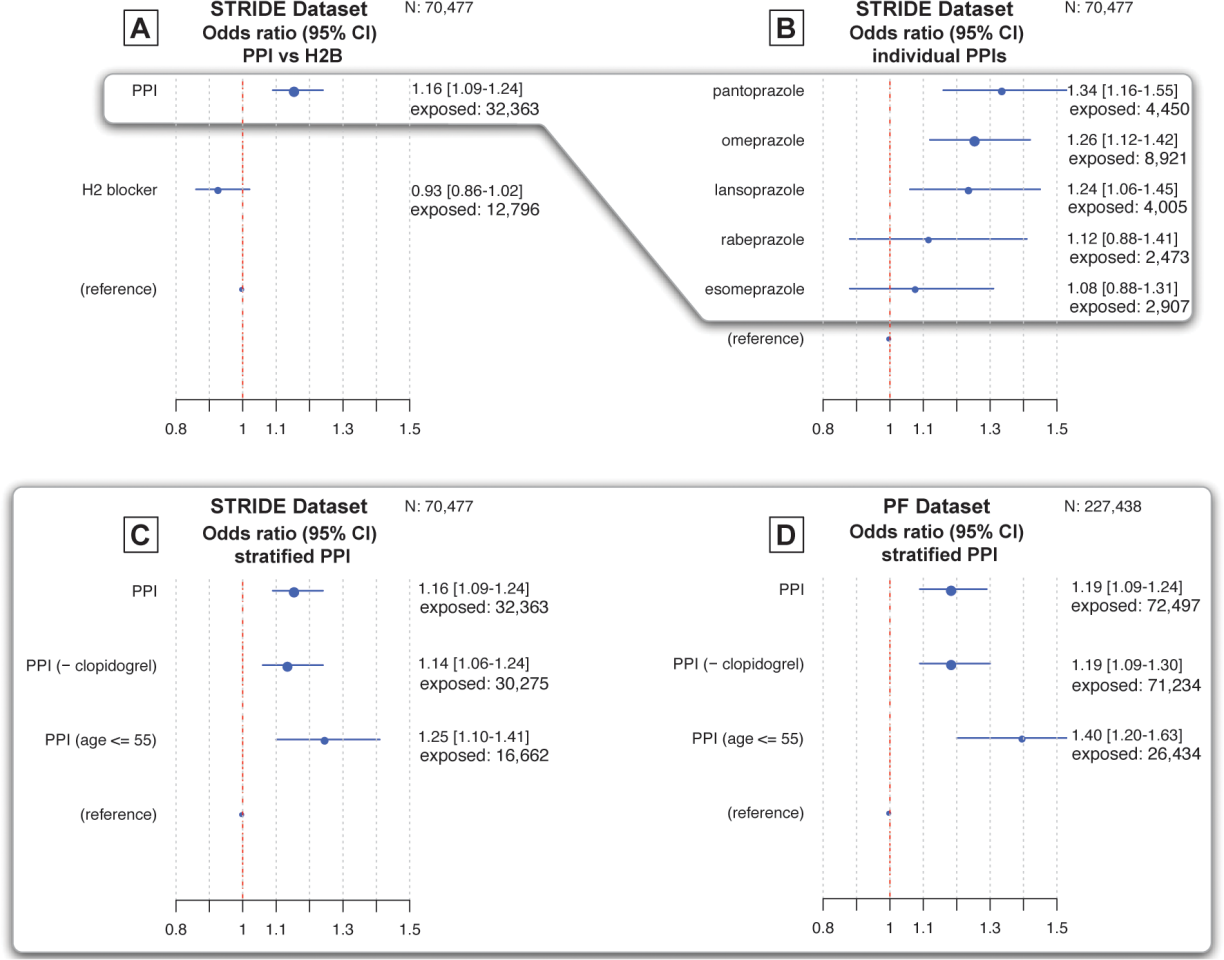
PPI use is associated with an increased risk for MI, regardless of age or clopidogrel use. No association is identified for H_2_ Blocker use: In the fig, the dotted red line represents the reference point indicating no elevated risk for myocardial infarction (MI). The odds ratio and 95% confidence interval for each exposure are indicated by a blue dot and blue line, respectively, which are also represented numerically to the right of each fig. The size of the dot is proportional to the exposure size of each group (see Table 1). Fig A, derived from STRIDE (N = 70,477), shows that PPIs have a class-level effect for MI in the general population of patients with GERD. By comparison, H_2_ blockers, an alternate treatment, have no association. Fig B breaks down the associations for each PPI individually. Figs C and D use stratification to show that the signals are corroborated in two independent datasets (STRIDE and Practice Fusion) and are robust in important subgroups. Fig C shows that, for the STRIDE dataset, when patients on clopidogrel are excluded, the associations are unchanged. Also, in lower-risk age groups for MI, the associations are still present. Similar trends are seen in these subgroups in the Practice Fusion (PF) dataset (N = 227,438) shown in Fig D.

For our data-mining method, a threshold of 1.0 on the lower bound of the 95% confidence interval of the adjusted odds ratios provides 39% sensitivity and 97.5% specificity in signaling an association—translating to a 3.5% false positive rate and a 61% false negative rate (making it a conservative test) [26]. Fig 1A shows that PPIs as a class (N = 32,363) are associated with MI with an adjusted odds ratio (AOR) of 1.16 (95% CI 1.09–1.24). Fig 1B shows the associations for each PPI individually. The strength of association varies slightly for each PPI, ranging from AOR 1.08 to 1.34.

### H2 blockers signal no association with MI

Given our hypothesis about the mechanism by which PPIs confer the increased risk, H_2_Bs (N = 12,796), an alternative treatment for GERD, are not expected to be associated with MI. Fig 1A confirms a lack of association (AOR 0.93; 95% CI 0.86–1.02).

### Associations are independent of clopidogrel use and age

Patients who take clopidogrel have often experienced a prior MI, and are likely to experience a second event. This population has been extensively studied [5, 7–10, 12, 15, 36]. A small fraction (~6%) of the PPI and H_2_B study groups are also on clopidogrel, which we examine separately for completeness. However, a known limitation of our data-mining methods, which focuses on first mentions, is the inability to pinpoint repeat occurrences of events [26], making it difficult to examine the clopidogrel treated group for a repeat coronary event. We addressed this by excluding patients with clopidogrel exposure.

Fig 1C, shows that the associations persist after excluding patients on clopidogrel and the association persists across age groups. Fig 1C shows that in patients not using clopidogrel (N = 30,275), the adjusted odds ratio does not differ markedly from general PPI use (AOR 1.14; 95% CI 1.06–1.24).

The results suggest that associations with MI are unlikely to be due to an interaction with clopidogrel, a surrogate for prior ACS history, which by itself would increase the likelihood of a second MI. In terms of risks related to age, Fig 1C also shows that the risks extend to individuals younger than 55 years old (N = 16,662), who are not a high-risk age group for MI. The mean age in the younger sub-group is 41.7, versus 54.5 in the general population, as shown in Table 2.

### Corroboration in an independent nationwide dataset

**Fig 2:**
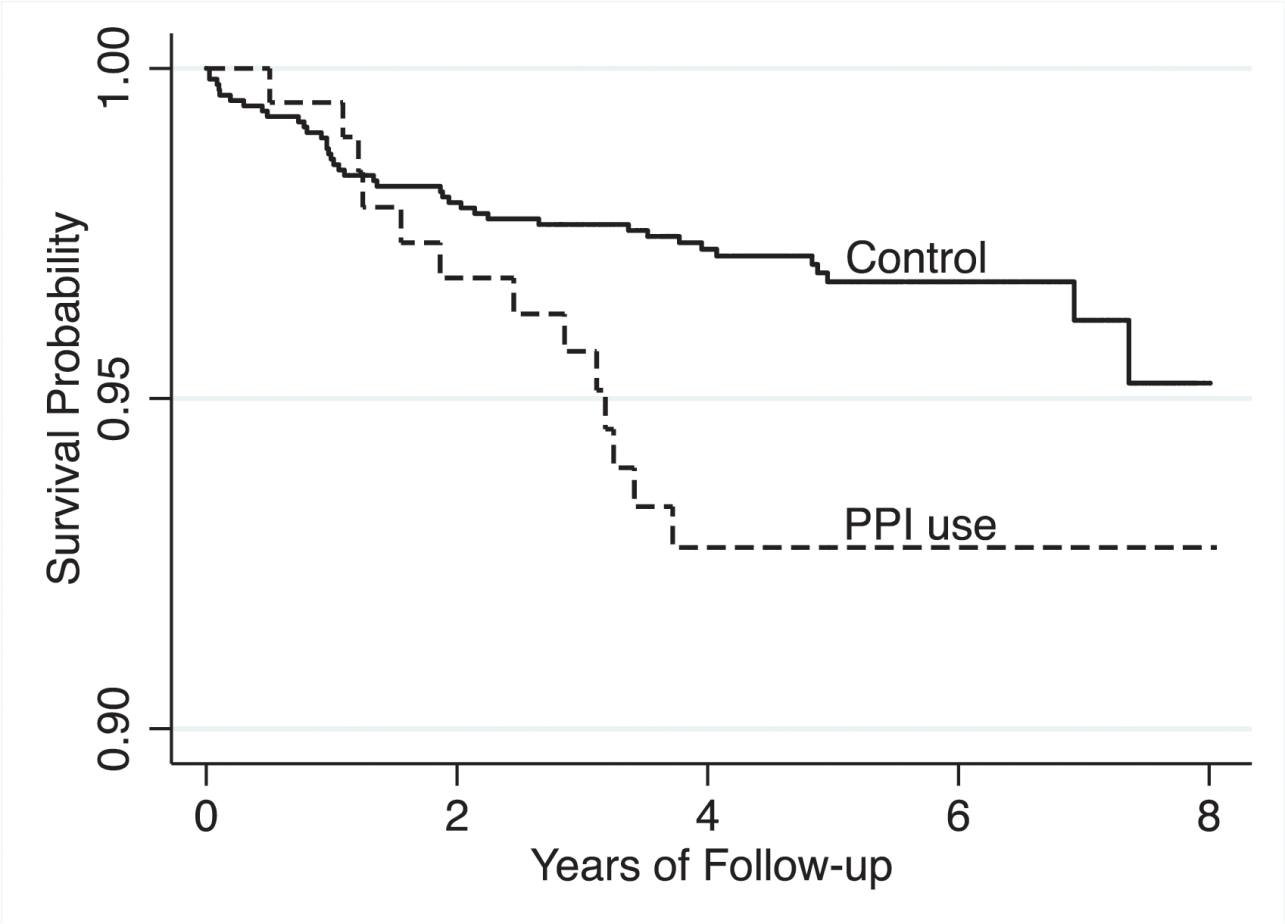
Survival plot from the prospectively followed GenePAD study confirms that PPI use is associated adverse outcome. The Kaplan–Meier curves in the fig show the survival probability from cardiovascular mortality according to PPI usage over an 8 years follow-up period in the ongoing Genetic Determinants of Peripheral Artery Disease (GenePAD) study. PPI usage is associated with a 2.22 fold (CI 1.19–4.16) increased risk of cardiovascular mortality, relative to controls in unadjusted Cox proportional hazards models.

Fig 1D shows a PPI class effect for an association with MI from an independent dataset. The PF dataset has a much larger and more heterogeneous set of GERD patients in the baseline population (N = 227,438) given the source of the data (see Table 4). The duration of coverage is shorter (2007 through 2012), with more patients entering the dataset only in recent years. As a result, balancing length of observation is difficult and our estimates of the method’s accuracy (described in the methods section) might not generalize. However, the results showed similar trends in the PF dataset data (AOR 1.19; 95% CI 1.09–1.30; Fig 2C and 2D) as were seen in STRIDE.

**Table 4:** Study group populations for the PF dataset.

		Before Matching			After Matching	
Study Group and Subgroups	N (GERD)	Exposed	Control	Match Ratio	Exposed	Control
Age > = 18 ‡	227,438					
PPI		74,516	152,922	1:2	72,497	152,922
(–) clopidogrel †		72,399	150,269	1:2	71,234	150,268
Age < = 45 ‡		17,508	29,454	1:2	13,287	29,453
Age < = 55 ‡		32,154	57,474	1:2	26,434	57,474
omeprazole		43,103	144,582	1:3	43,103	134,339
esomeprazole		8,078	129,049	1:5	8,078	44,521
pantoprazole		3,680	129,000	1:5	3,680	21,027
rabeprazole		998	128,570	1:5	998	5,808
lansoprazole		5,493	130,453	1:5	5,493	29,531

There are 227,438 patients with gastroesophageal reflux disease (GERD) who are at least 18 years old in this confirmation cohort. In all study groups other than the one in which clopidogrel patients are excluded, the populations are matched based on clopidogrel use and other covariates such as age, gender, race, length of observation, and number of unique drugs mentioned in the record as well as the number of unique disease concepts (as proxies for health status). The matching process attempts to achieve a ratio of 1:5 (exposure to control), but will settle for 1:3, 1:2, or 1:1 if there are not enough controls from which to draw. The length of observation in the PF dataset makes balancing variables difficult, so confounding cannot be entirely ruled out even after matching.

† patients on clopidogrel are excluded

‡ patients are restricted by age

### Survival analysis shows an association with cardiovascular mortality

In a separate analysis on the prospectively followed Genetic Determinants of Peripheral Arterial Disease [28, 29] (GenePAD) cohort—independent of our text-mining approach—there were 58 cardiovascular mortalities during a median follow-up period of 5.2 years (interquartile range, 4.1–6.3). Using a Cox proportional hazard model, an unadjusted analysis showed a 122% increased cardiovascular mortality risk among PPI users as measured by the hazard ratio (HR = 2.22; 95% CI 1.19–4.16; P = 0.013). This association persisted in the fully adjusted model (HR = 2.00; 95% CI 1.07–3.78; P = 0.031), which controlled for several cardiovascular comorbidities. Fig 2 shows a Kaplan–Meier curve representing the survival function from cardiovascular mortality for patients on PPIs versus controls. As with the text-mining analysis, no association was seen with H2Bs in either unadjusted (HR = 1.05; 95% CI 0.15–7.59; P = 0.962) or adjusted (HR = 1.00; 95% CI 0.14–7.26; P = 0.996) analyses.

### Associations are detectable as early as the year 2000

**Fig 3:**
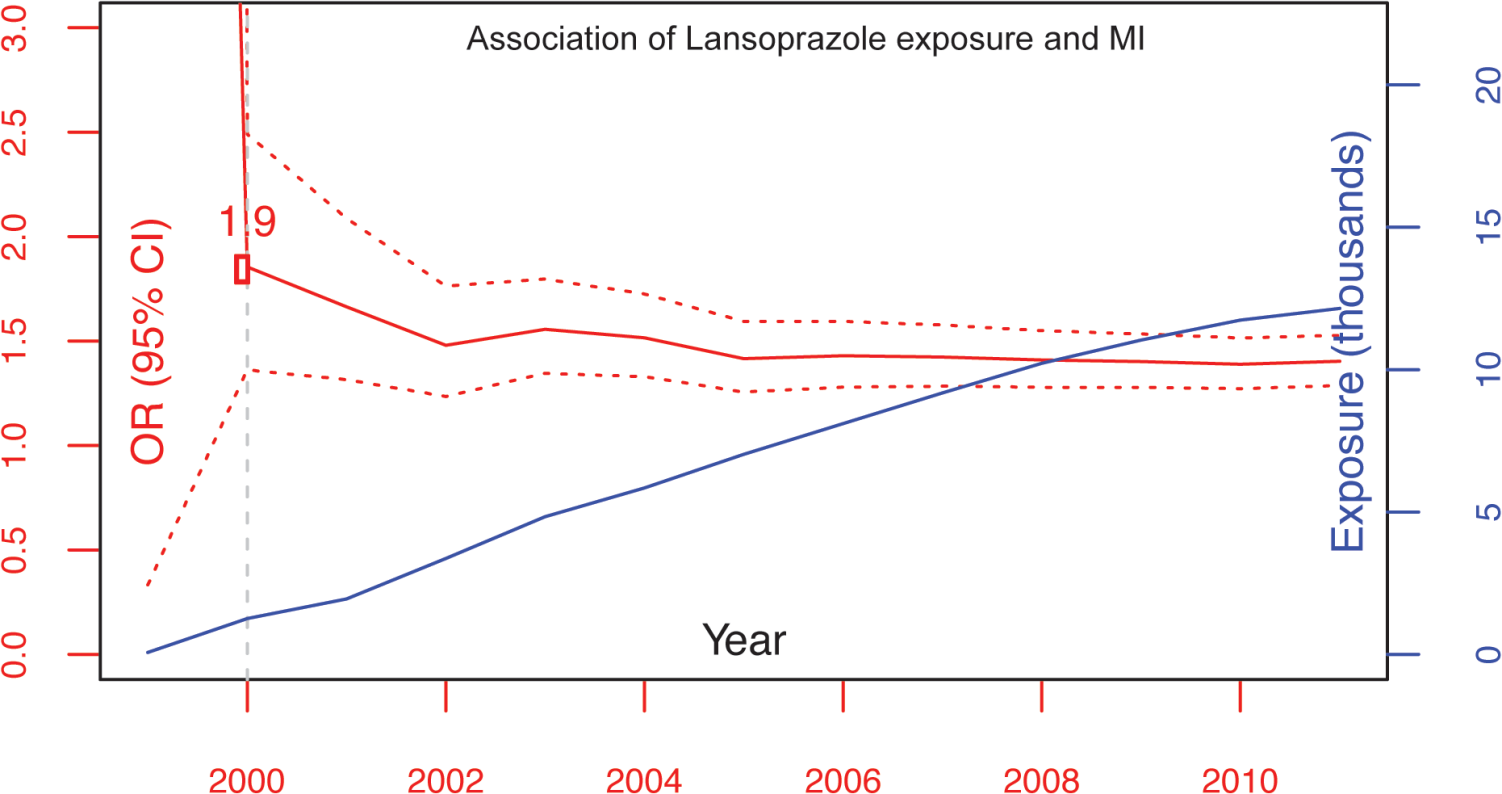
Cumulative risk and exposure plots reveal that pharmacovigilance algorithms could have flagged lansoprazole for monitoring as early as the year 2000. The x-axis is calendar year; the y-axis on the left is the unadjusted odds ratio; the y-axis on the right is the number of patients exposed. The solid red line is the point estimate of the odds ratio. The dotted red lines are the confidence intervals. The blue line is the number of patients exposed. Vertical lines mark the earliest detected signal—the year when the lower bound on the 95% confidence interval rises above 1.0. Signal detection algorithms on clinical notes would have flagged lansoprazole for monitoring as early as the year 2000.

The cumulative risk and exposure plot for lansoprazol shown in Fig 3 is based on the raw association estimates, which help to flag signals for early detection and monitoring as described in previous work.[26] Based on this plot, lansoprazole would have been flagged for monitoring in the year 2000 if we had such a data-mining system in place. As exposure data accumulates, the confidence intervals converge (note the narrowing 95% confidence intervals). Plots for the other PPIs are shown in the S2 Fig.

## Discussion

Our results demonstrate that PPIs appear to be associated with elevated risk of MI in the general population; and H2 blockers show no such association. The associations are independent of clopidogrel use or age-related risks and are seen in two large independent datasets and a prospective cohort. In particular, the association is seen outside of the high-risk populations previously examined, such as the elderly [38] or patients with ACS [2].

Our results are consistent with findings in the extensively-studied cohort of subjects with coronary artery disease (CAD) [5, 7, 12, 36], where PPIs have repeatedly been associated with adverse outcomes amongst patients receiving clopidogrel. [15] While two prospective studies in the post-ACS population failed to detect an association between PPI use and an increased risk of cardiovascular death, MI, or stroke [9, 10], the authors acknowledged that their results do not rule out a clinically meaningful difference in cardiovascular events due to use of a PPI.[10] In fact both studies included patients at a higher risk of MI than the general population, which may eclipse any potential harm conferred by PPIs due to competing risks. [38, 39] Based on the concern that PPIs could reduce the metabolism of clopidogrel to its active form, the FDA issued a warning about this possible drug-drug interaction in 2009 [40].

The current study suggests that the risk of PPIs may extend beyond previously studied high risk individuals. These findings confirm and extend the findings of Shih and colleagues, which suggested that PPIs were associated with short term cardiovascular harm amongst Taiwanese individuals [25], and are consistent with studies which have shown that PPIs may diminish the cardioprotective effects of drugs that do not depend on CYP2C19 activation, such as ticagrelor [7, 12, 13]. While it has been argued that this phenomenon might result from PPI-induced changes in drug absorption, we view this as a less likely possibility given that H_2_ blockers induce a similar reduction in gastric pH—without consistently increasing cardiovascular risk, as observed in each of three datasets studied here.[12] Other potential explanations for the observed association are that PPIs might impair cardiovascular hemodynamics or promote nutritional deficiencies. For example, PPIs have been reported to induce negative inotropic effects on myocardial tissue ex vivo, [41, 42] and to potentially increase the cardiovascular risk factor, homocysteine, by impairing the absorption of vitamin B12. [43, 44] However, population-based cohort studies have demonstrated a lack of excess mortality in patients with both ischaemic and non-ischaemic heart failure prescribed PPIs, [45] and consensus opinion is that PPIs are unlikely to cause a clinically relevant reduction in B12 levels in people on a normal diet, with otherwise normal gastrointestinal function [43].

Our observation that PPI usage is associated with harm in the general population—including the young and those taking no antiplatelet agent—suggests that PPIs may promote risk via an unknown mechanism that does not directly involve platelet aggregation. Accordingly, our recent molecular, cellular, physiological, and in vivo data [16] demonstrating that PPIs inhibit DDAH activity may explain how PPIs promote cardiovascular risk, and do so even in individuals not taking clopidogrel. DDAH, an enzyme necessary for cardiovascular health, metabolizes ADMA, an endogenous and competitive inhibitor of nitric oxide synthase (NOS).[46] Increases in plasma ADMA levels of as little as 10% are associated with increased risk of major adverse cardiovascular events.[19–24] We previously confirmed that PPIs inhibit purified DDAH enzyme using orthogonal assays. As a result, PPIs increased intracellular ADMA in cultured human endothelial cells by approximately 30%, increased serum ADMA levels in mice by approximately 20%, impaired endothelium-dependent vasodilation of isolated mouse aortae, and reduced the generation of nitric oxide by human saphenous vein segments obtained at the time of coronary artery bypass.[16] Taken together, these results provide a plausible mechanism for how PPI usage can manifest with dysregulation of vascular NOS, and therefore explain the association with increased risk of MI in the general population.

Our study is subject to several limitations. Most importantly, these observational data may be subject to confounding in multiple ways, and it is possible that PPI usage is merely a marker of a sicker patient population [13]. For example, we were unable to control for factors such as obesity and insulin resistance, and it may be that in some individuals PPIs were prescribed for angina that was misidentified as acid reflux. However, the observation that alternative heartburn medications such as H_2_ blockers were not associated with harm lends support to the concept that PPIs may specifically promote risk. Although our data-mining pipeline has high specificity and was validated to have high accuracy (89%), there is still a possibility that the association detected is a false positive. We also cannot account for over-the-counter PPI usage, or differences by drug dosage. We attempt to partially offset these limitations by including replication data from multiple sources (the community-based PF dataset, the tertiary-care Stanford dataset, and the prospective GenePAD study), and by adjusting for several cardiovascular covariates in the survival analysis. Nonetheless, we recognize that these findings are hypothesis generating, and a prospective randomized study in the general population (inclusive of both lean and obese individuals) is required before changing clinical practice. However, the number of subjects needed to detect harm among PPI users for MI is considerable, projected to be about 4,000 by Shih et al [25].

In conclusion, we use a novel analytical pipeline to associate PPI usage with risk of MI in the general population, independent of clopidogrel use. These findings, in conjunction with the preclinical results, necessitate additional investigation. Our work also puts forth an example use case of the learning health system on how multiple clinical data sources can be examined via data-mining to identify drug safety signals for further investigation. [47, 48]

## Supporting Information

S1 Table Indication, Drug, and Event definitions. For each clinical concept, a set of seed concept unique identifiers (CUIs) is used to generate a list of strings used to search through the clinical text.
(PDF)

S1 Fig Summary of the data-mining pipeline. To construct a contingency table, patients with gastroesophageal reflux disease (GERD) who were over 18 years old at the time of indication were identified and used to form the baseline population. The drugs of interest were PPIs, clopidogrel, and H2 blockers. The outcome was MI. The temporal ordering of the drug and outcome determined into which cell of a 2x2 contingency table each patient would be counted.
(PDF)

S2 Fig Cumulative risk and exposure plots for PPI–MI. Reveal that pharmacovigilance algorithms could have flagged omeprazole and lansoprazole for monitoring as early as the year 2000.
(PDF)
