# Supplementary material for: Proton Pump Inhibitor Usage and the Risk of Myocardial Infarction in the General Population
Source: PLoS One. 2015 Jun 10;10(6):e0124653. doi: 10.1371/journal.pone.0124653 (PMC4462578; doi:10.1371/journal.pone.0124653)
Supplement: S1 Table — For each clinical concept, a set of seed concept unique identifiers (CUIs) is used to generate a list of strings used to search through the clinical text. (PDF) [file pone.0124653.s001.pdf]

Supplementary Data S1: Indication, Drug, and Event defintions. For each clinical concept, a set of seed concept unique identifiers (CUIs) is used to generate a list of strings used to search through the clinical text. The frequency denoted by the number of patients (in STRIDE) having a mention of each string defines the sort order of each indication, drug or event. gerd - gastroesophageal reflux disease; mi - myocardial infarction

INDICATION	CUI	STRING	FREQUENCY
gerd	C0017168	reflux	116227
gerd	C0017168	gastroesophageal reflux	50570
gerd	C0017168	gerd	37206
gerd	C0017168	gastroesophageal reflux disease	33863
gerd	C0017168	acid reflux	13343
gerd	C0017168	esophageal reflux	4695
gerd	C0017168	gastric reflux	1615
gerd	C0017168	gastro-esophageal reflux	539
gerd	C0017168	gastric acid reflux	101
gerd	C0017168	gastroesophageal reflux disease with hiatal hernia	96
gerd	C0017168	hiatal hernia with gastroesophageal reflux disease	46
gerd	C0017168	reflux, gastroesophageal	33
gerd	C0017168	gastro-esophageal reflux disease	22
gerd	C0017168	gastro esophageal reflux	17
gerd	C0017168	oesophageal reflux	7
gerd	C0017168	gastresophageal reflux	6
gerd	C0017168	gastroesophageal reflux dis	6
gerd	C0017168	gastric regurgitation	5
gerd	C0017168	gastric acid reflux disease	4
gerd	C0017168	gerd1	4
gerd	C0017168	gastroesophageal reflux disease with apnea	2
gerd	C0017168	gastroesophageal reflux disease with ulceration	2
gerd	C0017168	gastroesophageal reflux disease without esophagitis	2
gerd	C0017168	reflux, gastric acid	1
gerd	C0017168	acid reflux, gastric	1

DRUG	CUI	STRING	FREQUENCY
dexlansoprazole	C2348248	dexilant	272
dexlansoprazole	C2348248	kapidex	234
dexlansoprazole	C2348248	dexlansoprazole	172
dexlansoprazole	C2348248	dexlansoprazole 60 mg	35
dexlansoprazole	C2348248	dexlansoprazole 30 mg	6
esomeprazole	C0937846	nexium	14590
esomeprazole	C0937846	esomeprazole	1304
esomeprazole	C0937846	esomeprazole 40 mg	143
esomeprazole	C0937846	esomeprazole magnesium	39
esomeprazole	C0937846	esomeprazole 20 mg	23
esomeprazole	C0937846	vimovo 500/20	2
lansoprazole	C0050940	prevacid	26671
lansoprazole	C0050940	lansoprazole	3572
lansoprazole	C0050940	lansoprazole 30 mg	1319
lansoprazole	C0050940	lansoprazole 15 mg	251
lansoprazole	C0050940	zoton	5
lansoprazole	C0050940	lanzoprazole	3
lansoprazole	C0050940	prevacid 30 mg enteric coated capsule	2
lansoprazole	C0050940	lansoprazole 30 mg disintegrating tablet	1
lansoprazole	C0050940	prevacid, 30 mg oral enteric coated capsule	1
lansoprazole	C0050940	lansoprazol	1
lansoprazole	C0050940	lansoprazole oral suspension	1
lansoprazole	C0050940	lansoprazole 15mg	1
lansoprazole	C0050940	ogastro	1
lansoprazole	C0050940	lansoprazole disintegrating tablet	1
omeprazole	C0028978	prilosec	35182
omeprazole	C0028978	omeprazole	17576
omeprazole	C0028978	omeprazole 20 mg	7754
omeprazole	C0028978	omeprazole 40 mg	1191
omeprazole	C0028978	omeprazole 10 mg	328
omeprazole	C0028978	zegerid	277
omeprazole	C0028978	omeprazole magnesium	84
omeprazole	C0028978	losec	57
omeprazole	C0028978	omeprazole 20 mg delayed release capsule	6
omeprazole	C0028978	omeprazole oral suspension	3
omeprazole	C0028978	omeprazole 20 mg oral tablet	3
omeprazole	C0028978	omeprazole 20 mg oral enteric coated capsule	2
omeprazole	C0028978	omeprazole 20 mg oral tablet, extended release	1
omeprazole	C0028978	omeprazole 40 mg delayed release capsule	1
omeprazole	C0028978	omep	1
omeprazole	C0028978	omeprazole 40 mg oral enteric coated capsule	1
omeprazole	C0028978	prilosec 20 mg enteric coated capsule	1
omeprazole	C0028978	omeprazole 4 mg/ml	1
omeprazole	C0028978	omeprazole 20mg ec cap	1
pantoprazole	C0081876	protonix	32497
pantoprazole	C0081876	pantoprazole	10014
pantoprazole	C0081876	pantoprazole 40 mg	2495
pantoprazole	C0081876	pantoprazole 20 mg	129
pantoprazole	C0081876	pantoprazole sodium	39
pantoprazole	C0081876	protonix 40 mg enteric coated tablet	1
pantoprazole	C0081876	pantoprazole granules	1
pantoprazole	C0081876	pantoprazole 2 mg/ml	1
rabeprazole	C0378482	aciphex	11996
rabeprazole	C0378482	rabeprazole	1092
rabeprazole	C0378482	pariet	16
rabeprazole	C0378482	rabeprazole sodium	13
rabeprazole	C0378482	rabeprazole na	3
rabeprazole	C0378482	rabeprazole sodium 20 mg	3

EVENT	CUI	STRING	FREQUENCY
mi	C0027051	myocardial infarction	39330
mi	C0027051	heart attack	22358
mi	C0027051	myocardial infarctions	2627
mi	C0948089	acute coronary syndrome	2277
mi	C0027051	myocardial infarction (mi)	2260
mi	C0155626	acute myocardial infarction	2131
mi	C0027051	acute myocardial infarction	2131
mi	C0027051	cardiogenic shock	1226
mi	C0155626	nstemi	1145
mi	C0027051	nstemi	1145
mi	C0027051	inferior myocardial infarction	757
mi	C0027051	myocardial infarct	737
mi	C0027051	non-st elevation myocardial infarction	554
mi	C0155626	non-st elevation myocardial infarction	554
mi	C0027051	inferior mi	549
mi	C0027051	anterior myocardial infarction	490
mi	C0027051	first myocardial infarction	356
mi	C0027051	anterior mi	334
mi	C0027051	inferior wall myocardial infarction	295
mi	C0340324	silent myocardial infarction	289
mi	C0027051	silent myocardial infarction	289
mi	C0027051	non-q wave myocardial infarction	286
mi	C0027051	old myocardial infarction	253
mi	C0027051	anterior wall myocardial infarction	176
mi	C0027051	old inferior myocardial infarction	133
mi	C0155626	acute inferior myocardial infarction	111
mi	C0027051	acute inferior myocardial infarction	111
mi	C0027051	postoperative myocardial infarction	94
mi	C0155626	acute anterior myocardial infarction	88
mi	C0027051	acute anterior myocardial infarction	88
mi	C0027051	anteroseptal myocardial infarction	85
mi	C0027051	non q wave myocardial infarction	66
mi	C0027051	posterior myocardial infarction	62
mi	C0027051	myocardial infarcts	60
mi	C0027051	subendocardial myocardial infarction	54
mi	C0027051	anterolateral myocardial infarction	44
mi	C0027051	lateral myocardial infarction	42
mi	C0027051	septal myocardial infarction	41
mi	C0155626	acute inferior wall myocardial infarction	35
mi	C0027051	cardiac infarction	35
mi	C0027051	acute inferior wall myocardial infarction	35
mi	C0027051	inferolateral myocardial infarction	33
mi	C0027051	old anterior myocardial infarction	32
mi	C0027051	apical myocardial infarction	28
mi	C0948089	acute coronary syndromes	26
mi	C0027051	myocardial stunning	25
mi	C0027051	subsequent myocardial infarction	24
mi	C0027051	acute anterior wall myocardial infarction	22
mi	C0155626	acute anterior wall myocardial infarction	22
mi	C0027051	posterolateral myocardial infarction	20
mi	C0155626	acute st elevation myocardial infarction	17
mi	C0027051	acute st elevation myocardial infarction	17
mi	C0027051	old myocardial infarct	11
mi	C0155626	acute inferior wall mi	11
mi	C0027051	acute inferior wall mi	11
mi	C0027051	myocardial infarction, inferior	10
mi	C0155626	myocardial infarction acute	8
mi	C0027051	myocardial infarction acute	8
mi	C0027051	myocardial infarction, inferior wall	7
mi	C0027051	inferior myocardial infarctions	7
mi	C0155626	acute anteroseptal myocardial infarction	7
mi	C0027051	shock, cardiogenic	7
mi	C0027051	acute anteroseptal myocardial infarction	7
mi	C0027051	acute anterolateral myocardial infarction	6
mi	C0027051	acute inferolateral myocardial infarction	6
mi	C0155627	acute anterolateral myocardial infarction	6
mi	C0155626	acute stemi	6
mi	C0155626	acute inferolateral myocardial infarction	6
mi	C0155626	acute anterolateral myocardial infarction	6
mi	C0027051	acute stemi	6
mi	C0027051	posteroinferior myocardial infarction	5
mi	C0027051	acute posterior myocardial infarction	4
mi	C0027051	myocardial infarction, anterior wall	4
mi	C0155626	acute posterior myocardial infarction	4
mi	C0027051	acute myocardial infarction, unspecified site	3
mi	C0155626	acute lateral wall myocardial infarction	3
mi	C0027051	infarct myocardial	3
mi	C0002965	intermediate coronary syndrome	3
mi	C0027051	acute lateral wall myocardial infarction	3
mi	C0027051	healed myocardial infarction	3
mi	C0155626	acute q wave myocardial infarction	3
mi	C0155626	acute myocardial infarction, unspecified site	3
mi	C0027051	acute q wave myocardial infarction	3
mi	C0027051	diaphragmatic myocardial infarction	2
mi	C0002965	impending infarction	2
mi	C0027051	anteroseptal myocardial infarctions	2
mi	C0027051	acute st segment elevation myocardial infarction	2
mi	C0155626	acute lateral myocardial infarction	2
mi	C0027051	acute lateral myocardial infarction	2
mi	C0155626	acute st segment elevation myocardial infarction	2
mi	C0027051	personal history of myocardial infarction	2
mi	C0155626	acute posterior wall mi	1
mi	C0027051	old posterior myocardial infarction	1
mi	C0155626	acute posterolateral myocardial infarction	1
mi	C0027051	true posterior myocardial infarction	1
mi	C0027051	acute posterior wall mi	1
mi	C0948089	syndrome, acute coronary	1
mi	C0155626	acute non-st segment elevation myocardial infarction	1
mi	C0027051	acute posterolateral myocardial infarction	1
mi	C0155626	acute anteroapical myocardial infarction	1
mi	C0155626	ami nos, unspecified	1
mi	C0948089	coronary syndrome, acute	1
mi	C0027051	infarction, myocardial	1
mi	C0027051	myocardial infarction old	1
mi	C0027051	acute non-st segment elevation myocardial infarction	1
mi	C0027051	acute anteroapical myocardial infarction	1
mi	C0027051	ami nos, unspecified	1
