# Supplementary material for: Proton Pump Inhibitor Usage and the Risk of Myocardial Infarction in the General Population
Source: PLoS One. 2015 Jun 10;10(6):e0124653. doi: 10.1371/journal.pone.0124653 (PMC4462578; doi:10.1371/journal.pone.0124653)
Supplement: S1 Fig — To construct a contingency table, patients with gastroesophageal reflux disease (GERD) who were over 18 years old at the time of indication were identified and used to form the baseline population. The drugs of interest were PPIs, clopidogrel, and H2 blockers. The outcome was MI. The temporal ordering of the drug and outcome determined into which cell of a 2x2 contingency table each patient would be counted. (PDF) [file pone.0124653.s002.pdf]

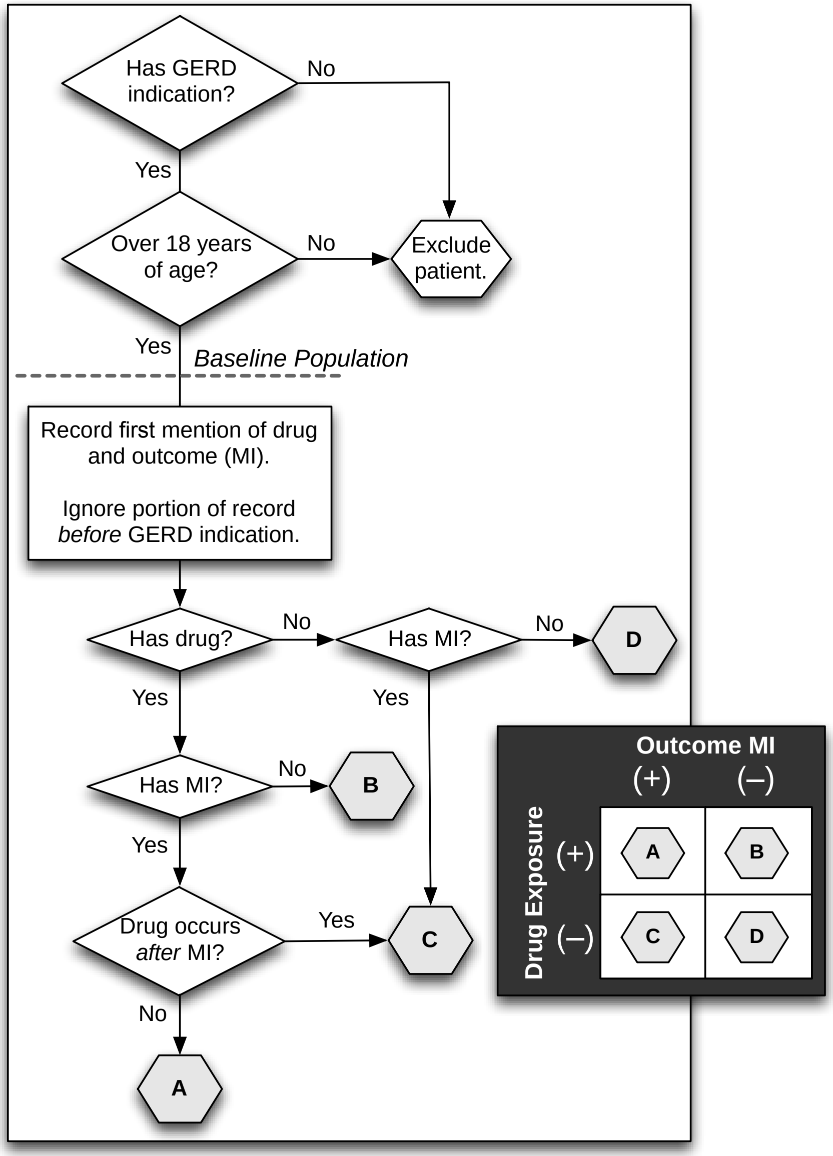

Supporting Information Figure S1: Summary of the data-mining pipeline

To construct a contingency table, patients with gastroesophageal reflux disease (GERD) who were over 18 years old at the time of indication were identified and used to form the baseline population. The drugs of interest were PPIs, clopidogrel, and H2 blockers. The outcome was MI. The temporal ordering of the drug and outcome determined into which cell of a 2x2 contingency table each patient would be counted. For example, patients over 18 with GERD who took omeprazole would be positive for treatment exposure for the PPI study group, and those who subsequently experience a MI would be counted in cell 'A'.
