# Supplementary material for: Proton Pump Inhibitor Usage and the Risk of Myocardial Infarction in the General Population
Source: PLoS One. 2015 Jun 10;10(6):e0124653. doi: 10.1371/journal.pone.0124653 (PMC4462578; doi:10.1371/journal.pone.0124653)

Supporting Information Figure S2

Cumulative risk and exposure plots for PPI–MI reveal that pharmacovigilance algorithms could have flagged omeprazole and lansoprazole for monitoring as early as the year 2000

For each plot, the x-axis is calendar year; the y-axis on the left is the unadjusted odds ratio; the y-axis on the right is the number of patients exposed. The solid red line is the point estimate of the odds ratio. The dotted red lines are the confidence intervals. The blue line is the number of patients exposed. Vertical lines mark the earliest detected signal — the year when the lower bound on the 95% confidence interval rises above 1.0. Signal detection algorithms on clinical notes would have flagged omeprazole and lansoprazole for monitoring as early as the year 2000.

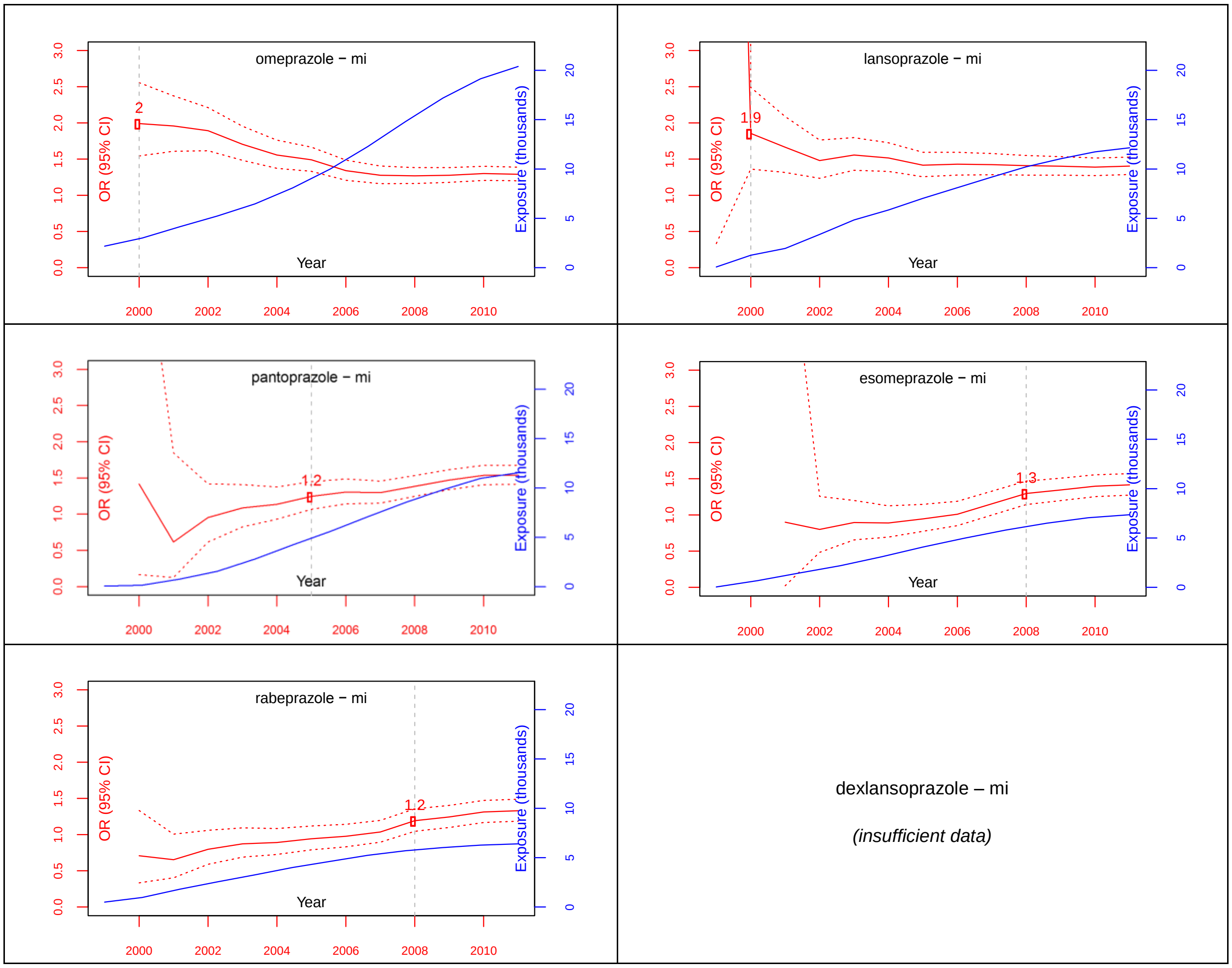

Supplement: S2 Fig — Reveal that pharmacovigilance algorithms could have flagged omeprazole and lansoprazole for monitoring as early as the year 2000. (PDF) [file pone.0124653.s003.pdf]
